# Establishment of Tree Shrew Animal Model for Kaposi’s Sarcoma-Associated Herpesvirus (HHV-8) Infection

**DOI:** 10.3389/fmicb.2021.710067

**Published:** 2021-09-16

**Authors:** Daoqun Li, Zulqarnain Baloch, Yang Zhao, Lei Bai, Xing Wang, Gang Wang, A-Mei Zhang, Ke Lan, Xueshan Xia

**Affiliations:** ^1^Faculty of Life Science and Technology, Kunming University of Science and Technology, Kunming, China; ^2^Institute of Basic Medicine, Shandong First Medical University and Shandong Academy of Medical Sciences, Jinan, China; ^3^State Key Laboratory of Virology, College of Life Sciences, Medical Research Institute, Wuhan University, Wuhan, China

**Keywords:** KSHV, Kaposi’s sarcoma, primary cells, tree shrews, animal model

## Abstract

Kaposi’s sarcoma-associated herpesvirus (KSHV) is the most common cause of Kaposi’s sarcoma (KS) and other malignant growths in humans. However, the lack of a KSHV-infected small animal model has hampered understanding of the mechanisms of KSHV infection, virus replication, pathogenesis, and persistence. This study was designed to explore the susceptibility of tree shrews as a possible KSHV-infected small animal model. A recombinant GFP (latent)/RFP (lytic)-positive rKSHV.219 strain was used to infect primary cells cultured from different tissues of tree shrews as an *in vitro* model and adult tree shrews as an *in vivo* model. KSHV latent nuclear antigen (LANA) and DNA were successfully detected in primary cells of tree shrews. Among them, tree shrew kidney epithelial cells (TSKEC) were the most susceptible cells to KSHV infection compared to other cells. KSHV genomic DNA, mRNA, and KSHV-specific proteins were readily detected in the TSKEC cultured up to 32 dpi. Moreover, KSHV DNA and mRNA transcription were also readily detected in the peripheral blood mononuclear cells (PBMCs) and various tissues of tree shrews infected with KSHV. Haematoxylin and eosin (HE) staining showed lymphocyte infiltration, lymphoid tissue focal aggregation, alveolar wall thickening, hepatocyte edema, hepatic necrosis in the spleen, lung, and liver of KSHV-infected animals. Additionally, immune-histochemical (IHC) staining showed that LANA or ORF62-positive cells were present in the spleen, lung, liver, and kidney of KSHV-infected tree shrews. Here, we have successfully established *in vitro* and *in vivo* KSHV latent infection in tree shrews. This small animal model is not only useful for studying the pathogenesis of KSHV *in vivo* but can also be a useful model to study transmission routes of viral infection and a useful platform to characterize the novel therapeutics against KSHV.

## Introduction

Kaposi’s sarcoma (KS)-associated herpesvirus (KSHV), also called human herpesvirus 8 (HHV-8), is a member of the gamma (γ)-herpesvirus subfamily of the genus *Rhadinovirus* (Prasad et al., 2012). KSHV infections are estimated to account for 34,000 new cancer cases globally. However, the KSHV prevalence substantially varies by race and geographic location (Wu et al., 2012). KSHV has the highest prevalence in sub-Saharan Africa (>90%), followed by the Mediterranean (20–35%), and United States, Northern Europe, Australia, Russia, Japan, as well as in China (<10%) (Katano and Sata, 2000; Gurtsevich et al., 2003; Rohner et al., 2014; Antonsson et al., 2015; Cesarman et al., 2019). KSHV establishes a latent infection in most infected individuals and is the causative agent of KS, primary effusion lymphoma (PEL), and multicentric Castleman’s disease (MCD). Clinical features of KS range from multifocal lesions of the spleen, lymph node, lungs, skin, and gastrointestinal tract. The four clinical subtypes of KS include classic, African endemic, immunosuppression-related, and AIDS-related KS. The classic form occurs on the lower extremities in elderly men of Mediterranean and Eastern European descent, the African endemic form involves visceral and lymphatic organ involvement occurring in young adults and children, and the immunosuppression-related form occurs in immunosuppressed patients also with diffuse involvement of the skin and visceral organs. The AIDS-related form occurs in HIV-1-infected individuals with diffuse involvement of the skin and internal organs (Friedman-Kien, 1981; Lidenge et al., 2019). Although substantial progress has been made in characterizing the virus, little is known about the mechanisms of latent KSHV infection development, virus replication, pathogenesis, and persistence (Yu X. et al., 2016).

Previously, several KSHV animal models have been developed, such as mice and marmosets (Chang et al., 2009; Ashlock et al., 2014). However, the genetic makeup, physiology, anatomy, and immune system of these models, particularly mice, are substantially distinctive compared to humans. Therefore, the mice model failed to recapitulate the viral replication, persistence, pathogenesis, oncogenic mechanisms, immune response, and cellular tropism of KSHV *in vivo* (Dittmer et al., 1999; Wang et al., 2014). Marmosets are valuable animal models for investigating the basic and applied research of KSHV infection. Still, the application of marmosets in biomedical research is very limited due to the high cost of production, low fertility rate, and serious ethical issues (Chang et al., 2009). Thus, there is a critical need for alternative animal models of KSHV that develop infections similarly to humans and thus provide a more appropriate animal model.

Tree shrew, a squirrel-like and rat-sized small mammal, and have a wide population throughout Southeast Asia and Southwest China. Whole-genome sequence analysis of Chinese tree shrews showed that Chinese tree shrews are genetically more similar to primates, and thus humans, than other animal models such as mice and rodents (Fan et al., 2013). Tree shrew has already been successfully established as a hepatitis B virus (HBV) (Walter et al., 1996), hepatitis C virus (HCV) (Amako et al., 2010), rotavirus (HRV) (Pang et al., 1983), hepatitis E virus (HEV) (Yu W. et al., 2016), herpes simplex virus (HSV) (Darai et al., 1978), and Epstein-Barr virus (EBV) (Wang Z. et al., 2017) animal model. Our group has already established Zika virus (ZIKV) infection *in vitro* in primary cells derived from different tree shrew tissues, and an *in vivo* model in tree shrews (Zhang L. et al., 2019; Zhang N. N. et al., 2019). The tree shrew has many distinctive characteristics, such as a short reproductive cycle, low production cost, high brain-to-body mass ratio, short gestation period, etc. Here, for the first time, we have successfully established KSHV infection by using rKSHV.219 virus with inserted green (GFP) and red (RFP) fluorescent protein genes (Myoung and Ganem, 2011) in both primary cells (cell-derived from various tree shrew tissues) and adult tree shrews.

## Materials and Methods

### Animals

Chinese tree shrews (*Tupaia belangeri Chinensis*) (F1 generation) were obtained from the experimental animal center of the Kunming Institute of Zoology (KIZ), Chinese Academy of Sciences (CAS). Tree shrews weighed 90 to 130 g and were 4–5 months old. All the animal-related experiments were strictly done in Animal Bio-safety level 2 laboratory according to the instructions of the Chinese Regulations of Laboratory Animals (Ministry of Science and Technology of the People’s Republic of China) and Laboratory Animal—Requirements of Environment and Housing Facilities (GB 14925-2010, National Laboratory Animal Standardization Technical Committee). This study was reviewed and approved by the Experimental Animal Ethics Committee at Kunming University of Science and Technology.

### Cell Lines and Primary Cell Culture

HEK293T cells (Human embryonic kidney epithelial cell) were cultured at 37°C, 5% CO_2_ in Dulbecco’s Modified Eagle medium (DMEM) (Gibco, United States), supplemented with 10% heat-inactivated fetal bovine serum (FBS), 1% non-essential amino acids, 100 units/mL penicillin, 100 μg/mL streptomycin, and 2 mM L-Glutamine. iSLK.219 cells were derived from SLK endothelial cells latently infected with the RTA-doxycycline inducible recombinant KSHV.219 virus which constitutively expresses puromycin *n*-acetyl-transferase and GFP while RFP is applied during lytic replication. iSLK.219 cells were cultured and passaged in DMEM containing 10% FBS, 4 μg/mL puromycin, 50 μg/mL hygromycin, and 50 μg/mL G418 (Sigma, United States) to maintain the rKSHV.219 episomal DNA. The primary cells were isolated from the kidney, liver, lungs, thoracic aorta, and peripheral blood of tree shrews after euthanization by injecting pentobarbital as previously described (Barron et al., 1990; Feng et al., 2017; Matsuda et al., 2017; Zhang L. et al., 2019). Subsequently, primary tree shrew lung epithelial cells (TSLEC) were cultured in DMEM (Hyclone, United States) supplemented with epidermal growth factor (EGF, 10 ng/mL) (PeproTech, United States). Primary tree shrew vascular endothelial cells (TSVEC) were cultured in Medium 200 (Gibco, United States) supplemented with low serum growth supplement (LSGS). Primary tree shrew peripheral blood mononuclear cells (TSPBMCs) were cultured in RPMI 1640 medium supplemented with interleukin-2 (IL-2, 10 ng/mL) (PeproTech, United States) and phytohemagglutinin-L (PHA-L, 2.5 μg/mL) Primary tree shrew kidney epithelial cells (TSKEC) were cultured in RPMI 1640 medium supplemented with EGF (10 ng/mL) rKSHV.219.

### Production and Purification

rKSHV.219 was multiplied by the induction of iSLK.219 cells with doxycycline as previously described (Myoung and Ganem, 2011). Briefly, iSLK.219 cells were induced in both the presence of doxycycline (Sigma, United States) and the absence of hygromycin, puromycin, and G418 (Sigma, United States) at approximately 90% confluency. Four days post-induction, cell supernatants were collected, cleared twice by centrifugation (2,500 × *g*, 30 min at 4°C), virus was passed through 0.45 μm pore size membrane filters, and then pelleted by ultracentrifugation (25,000 × *g*, 3 h at 4°C) using a CP-80WX (Hitachi, Japan). Virus pellets were then resuspended overnight in serum-free DMEM collection media and stored at −80°C. KSHV titer (GFU/mL) was determined in HEK293T cells by counting GFP-positive cells as described previously (Zhao et al., 2015).

Titer (GFU/mL) = [(green fluorescent cells/field)] × [(fields/well)/volume virus (mL) × (dilution factor)].

### Primary Cell rKSHV.219 Infection *in vitro*

Cultured tree shrew primary cells were infected by rKSHV.219 with a multiplicity of infection (MOI) of 10. The infection of cells was achieved by centrifugation at 1,250 × *g* at 25°C for 2 h after adding concentrated virus to the medium. After centrifugation, the cells were washed with PBS three times, and the fresh medium was replaced for further culture and observation. The HEK293T cells were used as positive control and primary cells from rats and mice as negative controls (Wang X. et al., 2017).

### Identification of TSKEC Using Immunofluorescence Assay (IFA)

Primary TSKEC were cultured in 10 cm dishes containing coverslips. After 24 h (h), coverslips were rinsed with PBS, fixed with 4% formaldehyde, permeabilized with 0.5% Triton X-100, blocked by corresponding antibodies, and then counterstained 1 μg/mL DAPI (Roche, Switzerland). For detection of expressed Keratin 18 (CK-18), TSKEC-containing coverslips were first incubated with mouse monoclonal antibodies against human CK-18 (Abcam, United States), then with FITC-conjugated goat polyclonal antibodies against mouse IgG (Abcam, United States). The cells’ visualization under Leica DMI3000B Manual Inverted Microscope and image quantification were carried out using NIS-Elements F 4.00.00 software (Leica, Germany).

### Tree Shrews rKSHV.219 Infection

For the animal infection experiment, a total of 16 tree shrews were used in this study, 13 (TS1–TS13) for challenge experiments (male:female = 7:6) and 3 (TS14-TS16) for control analysis (male:female = 1:2). The 13 tree shrews (TS1-TS13) were inoculated with rKSHV.219 5 × 10^7^ GFU by intravenous route and three animals (TS14-TS16) were inoculated with DMEM without serum as a negative control. Animals were given pentobarbital intramuscularly before blood sampling and euthanasia. Whole blood of each tree shrew collected at 3, 5, 7, 14, 21, 28, 35, 49, 56, 63, 77, 91, and 119 days post-infection (dpi) to detect viral DNA and RNA. PBMCs were isolated from whole blood by using peripheral blood mononuclear cell separation kit for mice (Solarbio, China) as per the manufacturer’s protocol. The isolated PBMCs were cultured in RPMI 1640 medium with IL-2 (10 ng/mL) (PeproTech, United States) and PHA-L (2.5 μg/mL). GFP and RFP were observed to evaluate latent and lytic infection of rKSHV.219 by using an inverted fluorescence microscope (Leica, Germany) at 24 h of cell culture. The spleen, liver, lung, thoracic aorta, and kidney were collected from dissected tree shrews at 7, 14, 21, 35, 49, 56, 63, 91, and 119 dpi.

### rKSHV.219 DNA and mRNA Quantification

Viral DNA was extracted from infected TSKEC, cell supernatant, peripheral blood, and tissues using TIANamp Virus DNA/RNA Kit (TIANGEN, China). Virus RNA was extracted from primary cells or animal tissues using Trizol buffer (Invitrogen, United States) according to the manufacturer’s instructions. Subsequently, the rKSHV.219 fragment was amplified with 50 ng DNA, specific primers ORFK9 (forward, 5′-GTC TCT GCG CCA TTC AAA AC-3′ and reverse, 5′-CCG GAC ACG ACA ACT AAG AA-3′), and SYBR Green Master Mix kit (Vazyme, China). The genomic DNA of rKSHV.219 was calculated by using a standard curve constructed with pcDNA3-ORFK9 DNA (Sun et al., 2014). Extracted RNA was used as a template for the generation of cDNA by using a High-Capacity cDNA Archive Kit (Applied Biosystems, United States). RT-PCR was performed with the SYBR Green Master Mix kit under the manufacturer’s protocol. The viral gene expression of LANA, RTA, ORF57, ORF59, and K8.1, and the expression of the inflammatory factors of IL-1β, IL-2, IL-6, IL-8, TNF-α, and IFN-γ were determined by quantitative RT-PCR using the primers (Supplementary Table 1). The housekeeping gene, β-actin, was used as an internal control, and uninfected tree shrews were used as reference controls. Relative gene expression levels were normalized to β-actin according to the comparative Ct (ΔΔCt) method. Results are expressed as mean ± standard deviations (SD). All samples were analyzed in duplicate in a 40-cycle reaction on an Applied Biosystems ABI 7500 machine. All experiments were repeated three times independently.

### Visualization of GFP/RFP-Positive Cells

rKSHV.219-infected TSKEC, TSH, TSLEC, TSVEC, TBPBMCs, primary rabbit and rat kidney, and HEK293T cells were observed at different time intervals using a Leica DMI3000B Manual Inverted Microscope. GFP fluorescence indicates latent infections, and RFP fluorescence indicates signifying lytic infection. Then, rKSHV.219-infected TSKEC and HEK293T were trypsinized, washed once with phosphate saline buffer (PBS), and resuspended in FACS buffer containing 4% FBS and 0.09% sodium azide. The expression of GFP and/or RFP-positive cells was examined on a flow cytometer (BD, United States). Data analysis was performed by FlowJo software and represented as either single result or the mean ± SD of 3 independent experiments. The percentage of infected cells was estimated by counting the number of infected host cells/number of total host cells. The results were expressed as the mean ± SD of infection percentage (%).

### Western Blot

All cells were lysed in radio immune precipitation assay (RIPA) buffer (Thermo, United States) supplemented with a complete protease inhibitor cocktail (Roche, Germany), and protein was quantified using a BCA protein assay kit (Beyotime, China). Purified proteins were resolved by 8–10% SDS-polyacrylamide gel electrophoresis (PAGE) and transferred onto a PVDF membrane (Bio-Rad, United States). Membranes were blocked in 5% non-fat dry milk in tris-buffered saline (TBS) with 0.1% tween (TBST) for at least an hour and then probed with indicated primary antibodies diluted in 5% defatted milk in TBST for 2 h on room temperature (RT), or overnight on 4°C with mouse monoclonal anti-LANA (GeneTex, United States), anti-ORF26 (Sigma, United States) and anti-β-actin (Abcam, United States) respectively, all at 1/1000 dilution. Blots were washed three times in TBST, and then probed with HRP-conjugated secondary antibody (Abcam, United States) diluted 1/5000 in TBST for 1 hat RT. Blots were washed three times in TBST, then subjected to chemiluminescence to capture images (Tanon, China).

### Immuno-Histochemical and Pathological Analysis

All tissue samples, including spleen, liver, lung, thoracic aorta, and kidney, were ground and fixed in 4% paraformaldehyde fix solution for 24 h (Sangon Biotech, China). Samples were dehydrated and embedded in paraffin, sectioned, and stained with hematoxylin and eosin (H&E). Immunohistochemistry for detecting protein expression of rKSHV.219 LANA and ORF62 was performed on 3 μm paraffin-embedded tissue sections using mouse anti-LANA monoclonal antibody (GeneTex, United States) and mouse anti-ORF62 monoclonal antibody (Sigma, United States), both at 1/100 dilution, according to the protocol of 2-step plus^®^Poly-HRP Anti-Mouse IgG Detection System and DAB detection system. Naïve tree shrew tissues were used as negative controls. Histological changes were observed by microscopy (Nicon, Japan).

### Statistical Analysis

All statistical data were analyzed with GraphPad Prism Software version 6.01 (GraphPad Software, United States). Survival curves were compared by the log-rank test. Statistical evaluation was performed with Student’s unpaired *t*-test. *P*-values of less than 0.05 were considered significant.

## Results

### Susceptibility of Tree Shrews Primary Cells to rKSHV.219 Infection

**FIGURE 1:**
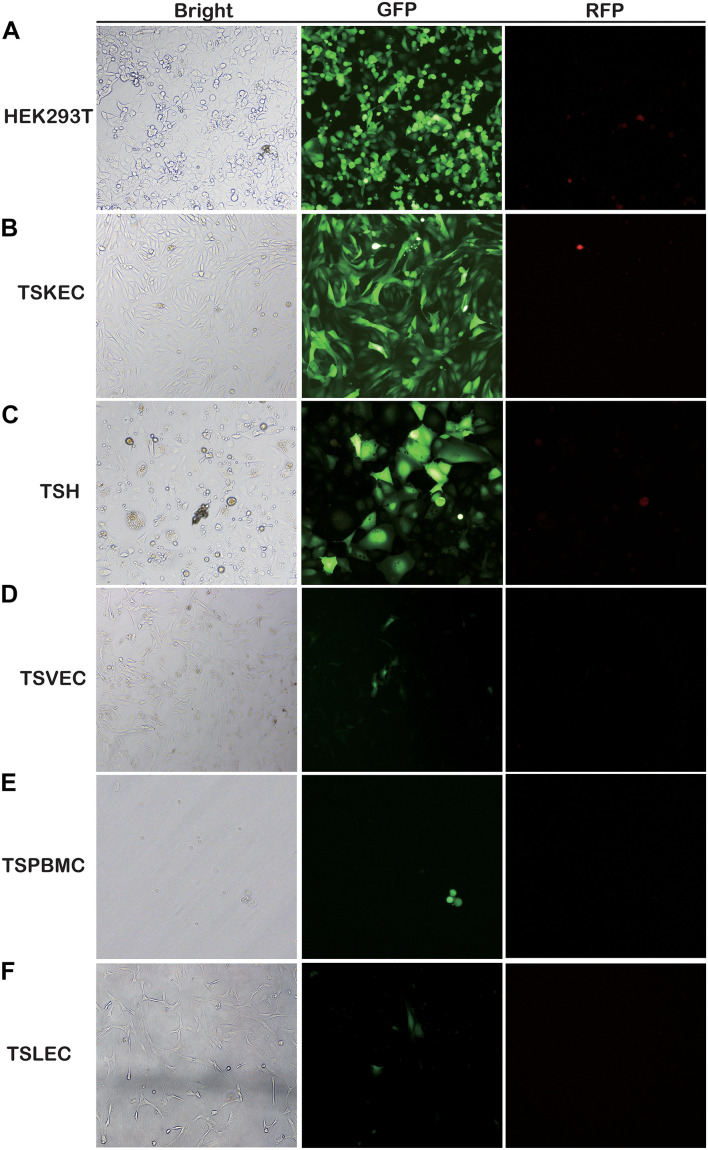
Screening for rKSHV.219-susceptible infected primary cells from tree shrews. Primary cells were infected with rKSHV.219 (MOI = 10). Bright field, GFP and RFP fluorescence images were taken at 48 h. **(A)** HEK293T. **(B)** TSKEC. **(C)** TSH. **(D)** TSVEC. **(E)** TSPBMCs. **(F)** TSLEC. Original magnification,10×.

Here, for the first time, we have investigated the susceptibility of various primary cells to rKSHV.219 infection. We found that TSKEC, TSH, TSVEC, TSPBMCs, and TSLEC are susceptible to rKSHV.219 infection (Figure 1 and Supplementary Figures 1A–F). Among these primary cells, TSKEC (96.9%), HEK293T (62.8%), and TSH (59.6%) were highly susceptible to rKSHV.219 infection compared to others (Supplementary Table 2). These results demonstrate that KSHV latent infection has been successfully established in multiple types of tree shrew primary cells, with a more efficient infection rate in primary TSKEC than in other cells.

### GFP and RFP Detection in rKSHV.219-Infected Primary TSKEC

Further, we compared the rKSHV.219 infectivity in TSKEC with the primary kidney cells of rats and rabbits. The latent infection rate of rKSHV.219 in TSKEC, rat, and rabbit kidney cells ranged from 82 to 97%, 18 to 39%, and 68 to 75%, respectively. However, the lytic infection rate was weaker than that of latent infection, with a range in TSKEC (1.04–22.78%), rat kidney cells (0.87–16.49%), and rabbit kidney cells (1.50–3.78%). Furthermore, TSKEC maintained long-term latent infection with decreased GFP expression at passages of cell culture with positive rates of 99% at p0, 50% at p3, 5—10% at, p5, 3–5% at p7, to <1% at p15. RFP-positive cells were detectable as early as p0 (Supplementary Figure 2).

We further characterized the rKSHV.219 infectivity in TSKEC at different times based on GFP and RFP expression pattern and virus-specific proteins detection. It revealed that GFP expression was initially detected as early as 6 h in TSKEC (Supplementary Figure 3A), gradually increased until 96 h, and then decreased with trends similar to HEK293T (Supplementary Figure 3B). Flow cytometry analysis showed that the GFP expression pattern varied from 69.71 to 97.1% (Supplementary Table 3). Overall, rKSHV.219 infectivity in TSKEC was similar to that of HEK293T (Supplementary Table 4). Additionally, we found that RFP expression can be detected as early as 24 h in TSKEC and then drops rapidly. RFP expression patterns varied from 64.84 to 0.92%, which indicates the disappearance of lytic infection. The percentage of RFP positive cells of HEK293T also decreased from 14.26 to 0.05%.

### Detection of rKSHV.219 Specific Protein in TSKEC

**FIGURE 2:**
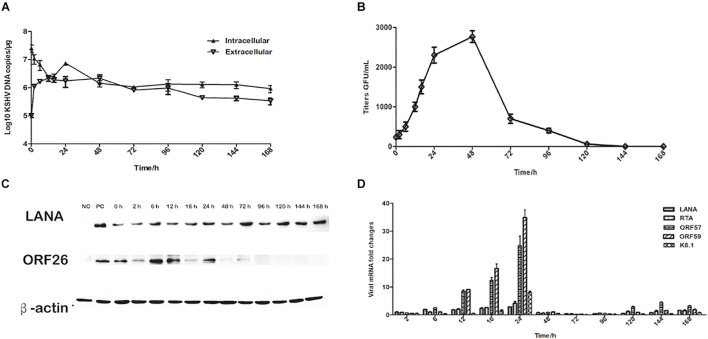
Characteristics of rKSHV.219 entry into the infected TSKEC (MOI = 10). **(A)** Intracellular and extracellular rKSHV.219 genome copies in TSKEC. **(B)** Titer of rKSHV.219 in TSKEC. **(C)** RT-q-PCR of rKSHV.219 transcripts in TSKEC. **(D)** The expression of KSHV LANA and lytic ORF26 proteins in TSKEC.

To further characterize rKSHV.219 infection dynamics in tree shrews, rKSHV.219 specific proteins were detected in the TSKEC. Our results showed that rKSHV.219 DNA replicated efficiently (DNA copies of 7.46 × 10^6^ copies/μg at 24 h) in TSKEC, with levels peaking (2.1 × 10^6^ copies/μg) at 48 h post-infection in the supernatant (Figure 2A). The titers reached 2.5 × 10^3^ GFU/mL at 48 h in the supernatant of the primary TSKEC (Figure 2B). The latent nucleoprotein expression of LANA had kept increasing within the first 48 h post-infection, and it then decreased from 72 to 168 h post-infection. During the infection period, the expression level of LANA nucleoprotein was always higher than that of lytic ORF26 capsid protein (Figure 2C). These results were consistent with that of HEK293T (Supplementary Figure 4).

Furthermore, we examined the expression of LANA, RTA, ORF57, ORF59, and K8.1 genes with quantitative RT- PCR. Our results showed that the mRNA of these proteins could be detected as early as 2 h post-infection and peaked at 24 h of post-infection (Figure 2D). The LANA mRNA was continuously detected over the infection period with a gradually increasing trend at the beginning of the infection, reaching the peak at 24 h of post-infection, which may be restricted by the response of the host cell. Comparatively, the expression of the lytic genes as RTA, ORF57, ORF59, and K8.1 were very low from 48 to 96 h post-infection. When calculated, the mRNA level of LANA is significantly higher than that of K8.1 (*p* = *0.048*).

**FIGURE 3:**
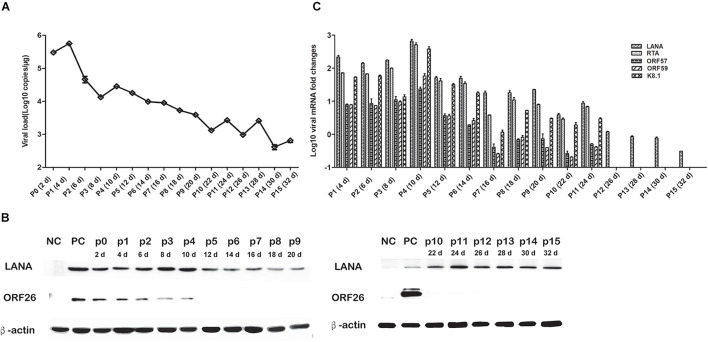
Cell passaged rKSHV.219 inTSKEC. **(A)** rKSHV.219 DNA copies in TSKEC. **(B)** rKSHV.219 transcripts in TSKEC. **(C)** rKSHV.219 latency protein LANA and lytic ORF26 expression in TSKEC (NC, negative control; PC, positive control; p0, passage 0).

To elucidate the persistence of rKSHV.219 long-term latent infection, the cells were trypsinized and passaged after every 2 days of post-infection till 32 days (16 passages). We found that the intracellular rKSHV.219 genome copy numbers fluctuated, with the viral genome peak appearing at p1 (Figure 3A). With the continued passage of time, the LANA mRNA level increased gradually and reached the maximum at p4. The LANA nucleoprotein maintained a moderate expression level but showed a downward trend after p10. The lytic ORF26 capsid protein is only detected in the early p4 generations (Figure 3B). Moreover, rKSHV.219 mRNA levels of LANA, RTA, ORF57, ORF59, and K8.1 could be persistently detected over the infection period and peaked at p4 (Figure 3C). Notably, the LANA mRNA level was higher than RTA, ORF57, ORF59, and K8.1 over the infection period.

### Replication of rKSHV.219 in Peripheral Blood of Tree Shrews

**FIGURE 4:**
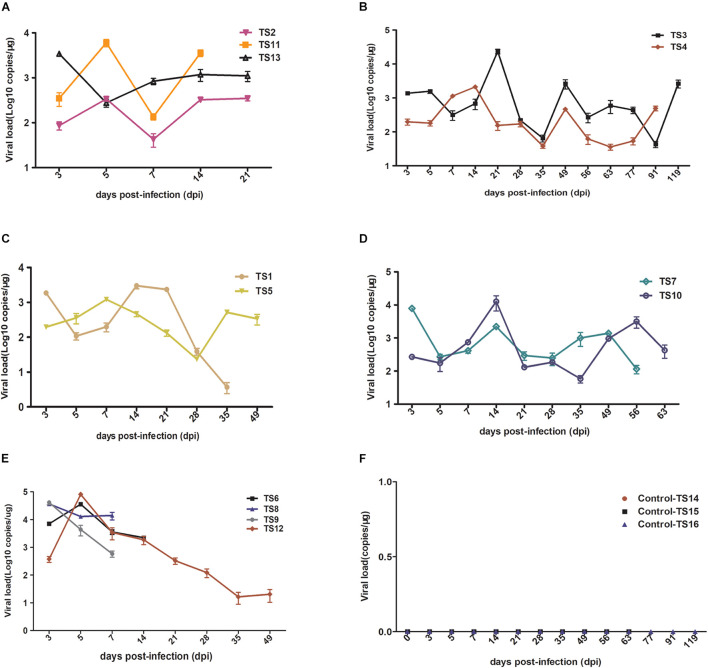
Detection rKSHV.219 copy number in peripheral blood of tree shrews TS1–13. TS14–16 were used as the negative control (NC); nothing was detected. **(A)** Intermittently increased in TS1, TS11, and TS13 at 14 and 21 dpi. **(B)** Intermittently increased in TS3 and TS4 at 91 and 119 dpi. **(C)** Intermittently increased in TS1 and TS5 at 35 and 49 dpi. **(D)** Intermittently increased in TS7 and TS10 at 56 and 63 dpi. **(E)** Transiently increased in TS8, TS9, TS6, and TS12 at 7, 14, and 49 dpi, respectively. **(F)** Negative control of TS14, TS15, and TS16 at 21, 63, and 119 dpi, respectively.

To further characterize rKSHV.219 infection dynamics in tree shrews, blood samples were collected from 16 animals and subjected to virological assays. Viremia was detected in all 13 animals (100%, 13/13) injected with rKSHV.219. Among 13 animals, the rKSHV.219 copy number increased intermittently in 9 animals (Figures 4A–D). However, the KSHV copy number only increased transiently in the remaining four tree shrews, and the peak of viremia appeared at 3 dpi (TS8 and TS9) and 5 dpi (TS6 and TS12) (Figure 4E).

Among these 13 infected tree shrews, the copy number of rKSHV.219 was the highest in TS12 (8 × 10^5^ copies/μg). All control animals (TS14–TS16) were negative (Figure 4F). PBMCs were collected from all infected tree shrews at the time of euthanization and cultured for 24 h. TS1, TS3, and TS5 (23.08%, 3/13) were GFP-positive (Supplementary Figure 5). Approximately 1–2/10^6^ lymphocytes were GFP-positive and RFP-negative, suggesting latent KSHV infection has developed. In contrast, no GFP/RFP-positive cells were detected in the negative control.

RT-PCR analysis showed that the LANA mRNA level was high in TS1, TS2, and TS6 (Supplementary Table 5). The LANA mRNA level was intermittent in TS3, TS4, TS5, TS7, TS10, and TS12 (Supplementary Table 5). However, the LANA mRNA levels in TS8, TS9, TS11, and TS13 were detected during the early stage of infection, then disappeared. For the lytic stage, the RTA mRNA level was inconsistent (TS3–TS7). For other lytic genes, the ORF57, ORF59, and K8.1 mRNA were mainly seen in the initial phase of infection (≤5 dpi) and then only showed intermittently, except for TS3 and TS12 (Supplementary Table 5).

### Pathological Changes of rKSHV.219-Infected Tree Shrews Tissues

**FIGURE 5:**
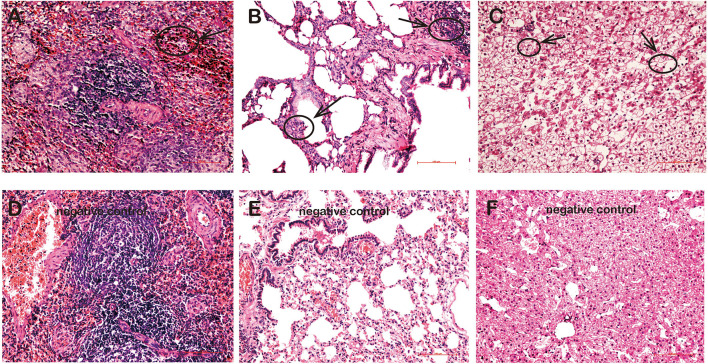
Pathological changes in the tissues of rKSHV.219-infected tree shrews. **(A)** Inflammatory cell infiltration and hyperplasia in the spleen of TS3 at 119 dpi. **(B)** Extensive inflammatory lymphocyte infiltration and alveolar wall hyperplasia in the lung of TS7 at 56 dpi. **(C)** Hepatic edema and hepatic sinus expansion in the liver of TS3 at 119 dpi. Panels **(D–F)** were negative control of spleen, lung, and liver, respectively. Original magnification, 20×.

Pathologically, various degrees of lymphocyte infiltration, alveolar wall hyperplasia, and degeneration were observed in tissues of rKSHV.219-infected tree shrews. Overall, the lymphocyte infiltration was found in spleens of four animals (TS3, TS5, TS7, and TS10), but lymphocyte infiltration was visible only in TS3 (Figure 5A). Pathological changes were also found in the lungs of six rKSHV.219-infected animals (TS4, TS7, TS9, TS10, TS11, and TS13), with the most distinct appearance being seen in TS4. Simultaneously, we also found local alveolar wall hepatocyte edema thickening in the lungs of six animals (TS4, TS7, TS9, TS10, TS11, and TS13) (Figure 5B). In addition, hepatic sinus, hepatocyte edema degeneration, and sinusoidal dilatation were observed only in the TS3 liver (Figure 5C). No pathological change cells were detected in the kidney and thoracic aorta of rKSHV.219-infected tree shrews. In contrast, no pathological changes were found in the spleen (Figure 5D), lung (Figure 5E), and liver (Figure 5F) of negative controls.

### rKSHV.219 Tissue Tropism in Tree Shrews

**FIGURE 6:**
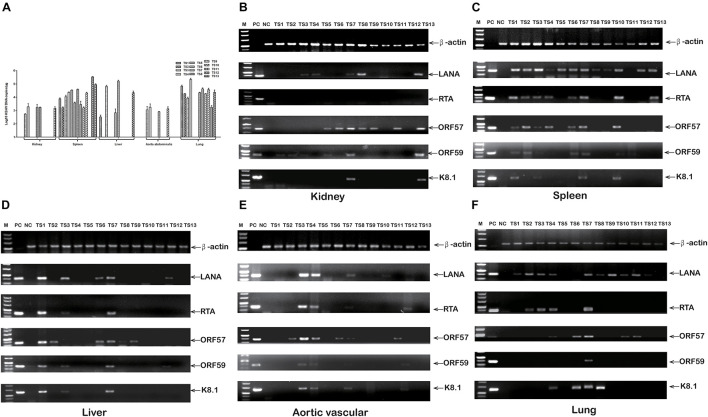
rKSHV.219 DNA copies and mRNA transcripts in multiple tissues of TS1-13 tree shrews. **(A)** rKSHV.219 DNA copies in the different tissues. **(B)** rKSHV.219 mRNA transcripts in the kidney. **(C)** rKSHV.219 mRNA transcripts in the spleen. **(D)** rKSHV.219 mRNA transcripts in liver. **(E)** rKSHV.219 mRNA transcripts in thoracic aorta. **(F)** KSHV mRNA transcripts in lung. (M, DL500 DNA Marker; NC, negative control; PC, positive control).

To elucidate rKSHV.219-infected tree shrews tissue tropism, virus DNA, and mRNA, were further detected. The detection rate of rKSHV.219 DNA was as high as 92.31% (12/13), 76.92% (10/13), 38.46% (5/13), 38.46% (5/13), and 30.77% (4/13) in the spleen, lungs, kidney, liver, and thoracic aorta of tree shrews, respectively (Supplementary Table 6). Among them, the rKSHV.219 copy number was high in the spleen of TS12 (2.4 × 10^5^ copies/μg) (Figure 6A). The LANA mRNA was found in the kidneys of 5 animals (TS3, TS4, TS7, TS8, and TS13) (Figure 6B), spleens of 12 animals (TS1-TS10, TS12, and TS13) (Figure 6C), livers of 5 animals (TS1, TS3, TS6, TS7, and TS12) (Figure 6D), thoracic aortas of 4 animals (TS3, TS4, TS7, and TS10) (Figure 6E) and lungs of 10 animals (TS1–TS4 and TS7–TS12) (Figure 6F). Lytic RTA mRNA was detected in 10 spleens (TS1–TS4, TS6, TS7, and TS10–TS13), three livers (TS1, TS3, and TS7), three thoracic aortas (TS3, TS4, and TS7), and four lungs (TS2–TS4 and TS7). Lytic ORF57 mRNA was found in nine spleens (TS1–TS4, TS6, TS7, and TS10–TS12), three livers (TS1, TS6, and TS7), three thoracic aortas (TS3, TS4, and TS7), four lungs (TS4, TS7, TS10, and TS11), and three kidneys (TS7, TS8, and TS13). Lytic ORF59 and K8.1 mRNA levels were simultaneously detected in two kidneys (TS8 and TS13), four spleens (TS1, TS3, TS7, and TS10), three livers (TS1, TS3, and TS7), two thoracic aortas (TS3 and TS4), and one lung (TS7).

**FIGURE 7:**
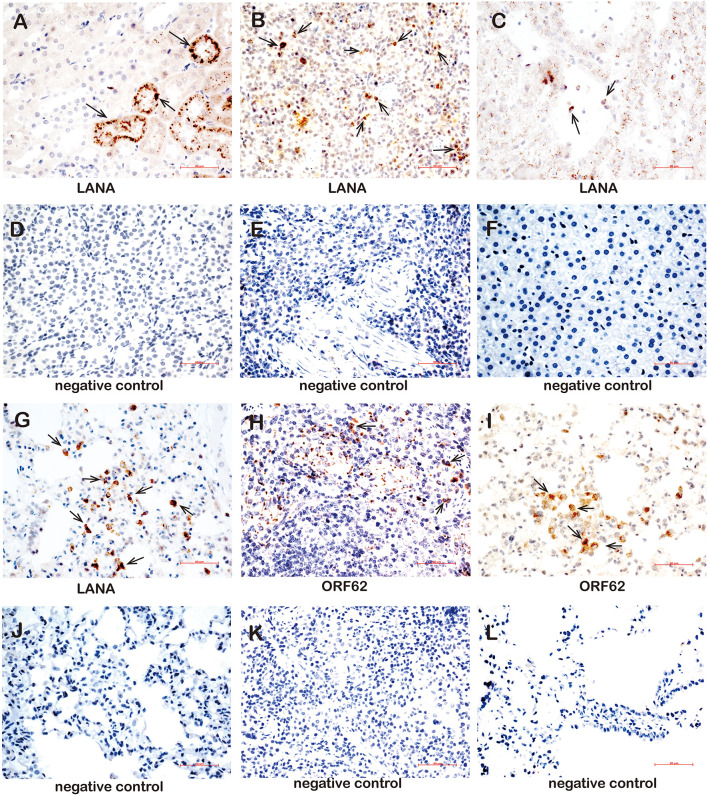
Immunohistochemistry detection in tissues of rKSHV.219-infected tree shrews. **(A)** The kidney epithelial cells of the TS3 kidney were nuclear staining positive for LANA at 119 dpi. **(B)** The lymphocytes of the TS4 spleen were nuclear staining positive for LANA at 91 dpi. **(C)** Hepatocytes of TS3 liver were nuclear staining positive for LANA at 119 dpi. Panels **(D–F)** were LANA negative control of kidney, spleen, and liver, respectively. **(G)** The alveolar tissues and lung epithelial cells of TS11 lung were nuclear staining positive for LANA at 14 dpi. **(H)** The lymphocytes of the TS3 spleen were cytoplasm staining positive for ORF62 at 119 dpi. **(I)** The alveolar tissues and lung epithelial cells of TS7 lung were cytoplasm staining positive for ORF62 at 56 dpi. **(J)** was LANA negative control of lung. Panels **(K,L)** were ORF62 negative control of spleen and lung, respectively. Original magnification, 40×.

Furthermore, the LANA and lytic ORF62 protein were detected by using immune-histochemistry (Supplementary Table 7). The LANA protein was detected in the kidneys of three animals (TS3, TS4, and TS8) (Figure 7A), in the spleens of eight animals (TS1, TS3, TS4, TS5, TS7, TS8, TS9, and TS10) (Figure 7B), the liver of one animal (TS3, Figure 7C), and the lungs of five animals (TS4, TS7, TS9, TS10, and TS11) (Figure 7D). In contrast, no LANA protein was detected in the kidneys (Figure 7E), spleen (Figure 7F), liver (Figure 7G), and lungs (Figure 7H), respectively. Lytic ORF62 protein was positive only in the TS3 spleen (Figure 7I) and TS7 lung (Figure 7J). In contrast, no ORF62 protein was found in the spleen (Figure 7K) and lungs (Figure 7L) of the control animals.

### The Immune Response of Tree Shrews to rKSHV.219 Infection

**FIGURE 8:**
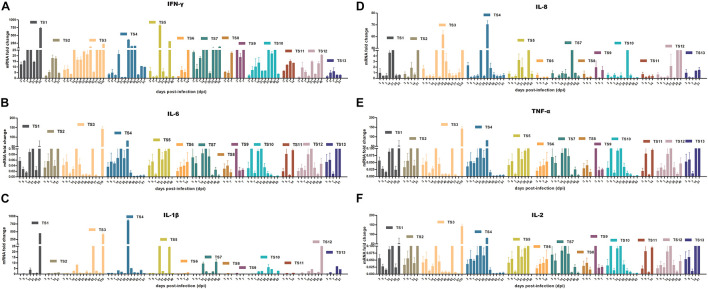
KSHV induces an innate antiviral response in TS1–13 tree shrews *in vivo*. **(A)** IFN-γ. **(B)** IL-6. **(C)** IL-1β. **(D)** IL-8. **(E)** TNF-α. **(F)** IL-2.

To determine whether rKSHV.219 infection induces an innate antiviral immune response, we further analyzed the expression of key antiviral immunity-related cytokines in rKSHV.219-infected tree shrews. The rKSHV.219-infected tree shrews induced a stronger antiviral response than mock groups *in vivo*. Particularly, the mRNA of IFN-γ, which may inhibit cell growth and protect against viral infection, showed a high level with a significant increase of >1,000-fold in all rKSHV.219-infected tree shrews in all infection periods (Figure 8A). The level of mRNA of IL-6, which may enhance angiogenesis through the induction of VEGF, fibroblast growth factor synthesis, and cell inflammatory response, was highly induced as early as 3 dpi (Figure 8B). The mRNA level of IL-1β, IL-8, TNF-α, and IL-2 was down-regulated (<1-fold) in the early stages of infection (Figures 8C–F). However, the levels of multiple inflammatory cytokines, such as IL-1β, IL-2, IL-8, and TNF-α were slowly elevated after 14 dpi. These results demonstrated that rKSHV.219-infected tree shrews could generate a robust innate immune response to rKSHV.219 infection.

## Discussion

KSHV animal and cell models are effective tools for studying the proliferation and pathogenic mechanism of the virus, screening antiviral compounds, and developing preventive vaccines (Parsons et al., 2004; McAllister and Moses, 2007). Tree shrew is a novel non-human primate animal model that is becoming widely used as an animal model for different viral infections (Vercauteren et al., 2014). For the first time, we have described KSHV infectivity in the primary cell-derived from different tissues of tree shrew as an *in vitro* model and successfully developed tree shrew as KSHV model by tail intravenous injection of rKSHV.219 strains. Different rKSHV.219-infected primary cells showed a distinct and prominent GFP expression, consistent with occurrences of rKSHV.219 infection in corresponding tissues of tree shrews *in vivo*. Notably, GFP and LANA nucleoprotein expression were significantly higher in TSKEC than in other primary cells.

Additionally, the human HEK293T cells could actively support rKSHV.219 infection, which is consistent with previous research (Bechtel et al., 2003). We also found that TSKEC had higher infection efficiency compared to primary kidney cells of rabbits and rats. Compared to previous reports, infection efficiency in TSKEC was higher than primary human umbilical vein endothelial (HUVEC) cells (Ciufo et al., 2001), primary B cells (Mesri et al., 1996), Chinese hamster fibroblasts (CHO), and quail fibroblasts (QT-6) cell lines (Bechtel et al., 2003), and slightly lower than rat primary mesenchymal precursor cells (MM cells) (Jones et al., 2012). Further, we found that rKSHV.219 latent infection was persistent with increased passage numbers. The current study-based developed rKSHV.219 latent infection was successfully maintained for a longer time than human telomerase-immortalized microvascular endothelial cells (16 generations *vs.* 10 generation*s*) (Lagunoff et al., 2002).

Next, a total of 13 mature tree shrews were infected with 5 × 10^7^ PFU of rKSHV.219 by the intravenous tail route. In this study, all animals were regularly monitored for clinical signs and symptoms throughout the experiment period. They developed an intermittent and transient type of infection similar to phenomena reported in different populations (Olp et al., 2016). Further, we used RT-PCR to detect mRNA levels in the peripheral blood samples. The LANA transcripts were detected throughout the infection period, while, immediate early lytic ORF50, early lytic ORF57, and ORF59 transcripts were detected at a very low level. These results indicated that latent infection was established in tree shrews in line with data reported by Wang et al. (2014). Additionally, rKSHV.219 infection has been successfully established in murine B cells, macrophages, NK cells, and, to a lesser extent, dendritic cells (Bechtel et al., 2003). KSHV-transformed MM (KMM) cells efficiently induce tumors in nude mice that are distinct from human tumors. This tropism preference may be caused by the difference in animal species (Jones et al., 2012). rKSHV.219 infection was detected in various organs, including the spleen, liver, lungs, and kidney of tree shrews, which agreed with murine herpesvirus 68 (MHV-68) in mice with similar tissue tropism, pathological features, and virus persistence. But MHV-68 infection does not associate with KS or related diseases (Sunil-Chandra et al., 1992). Moreover, our results also indicated that the lungs, thoracic aortas, and kidney tissues were additional infection and replication sites of KSHV.

Previously, a prominent inflammatory infiltrate was found during the early phase of KS lesions development PEL and MCD (Mesri et al., 2010). Furthermore, KSHV-infected human patients’ lungs exhibited lymphocytic interstitial pneumonia, follicular bronchiolitis, and lymphoid hyperplasia, and their livers showed necroscopic changes and hepatomegaly (Park and Levinson, 2010; Van Leer-Greenberg et al., 2017). This study also found the spleens, lungs, and livers of rKSHV.219-infected animals presenting massive inflammatory cell infiltration, cellular edema, follicular bronchiolitis, thickening, and necrosis. Most of the pathological changes were accompanied by the lytic gene K8.1 expression. These are the lymphocytes, epithelial, and hepatocytes cells, where viral lytic gene expression and local tissue factors could mediate their effects and expansions. Consistent with previous studies (Wu et al., 2006; Park and Levinson, 2010; Van Leer-Greenberg et al., 2017), we showed that KSHV infection occurred in lymphocytes, epithelial cells, and sinusoidal endothelial cells from tree shrews. Among various tissues, the spleen was the candidate target tissue KSHV infection, and KSHV enters multiple tissues and organs of the tree shrew through lymphocyte carriers to promote its spread. However, it is difficult to determine whether lymphocytes were B cells or T cells because of the absence of tree-shrew-specific reagents. Besides, LANA- or ORF62- positive cells were also detected in spleens, lungs, livers, and kidneys. This will give aid us in understanding the pathogenesis of KSHV in future research.

KSHV infection is characterized by a strong immune reaction or antiviral response and abundant expression of inflammatory cytokines (Hussein et al., 2017). These cytokines play a major role in the development and maintenance of KS infection and are also involved in the clinical manifestations of the disease. Patients with all forms of KS and individuals at risk of KS, including HIV-1-infected individuals and older people of Mediterranean origin, show CD8^+^ T cell and Th1-type activation and increased peripheral blood levels of IL-1, IL-6, TNF-α, and IFN-γ, or show an oligoclonal expansion of CD8^+^ T cells (Ensoli et al., 2000). We dynamically analyzed the key immunity-related cytokines expression in rKSHV.219-infected tree shrews. Our data revealed a strong antiviral immune defense. In detail, mRNA expression of IFN-γ and IL-6 showed a significant and rapid increase. IFN-γ high production plays a central role in hosting protection against KSHV infection involving increasing CD4^+^ and CD8^+^ lymphocytes, explaining low KSHV DNA levels (Guedes et al., 2008). The mRNA expression of IL-1β, IL-2, IL-8, and TNF-α was suppressed in the early stages (7 dpi), then began to increase (14 dpi). Herein, the high levels of IFN-γ expressed strengthened the ability of the immune system to achieve virus clearance in tree shrews (TS6, TS8, TS9, and TS12) and transiently affected the infection status. Thus, it appears that KSHV has multiple strategies to interfere with detection by the host immune system to support long-term dormant infection *in vivo* in tree shrews.

Although rKSHV.219 DNA replication, mRNA expression, protein detection, a small number of lymphocytes were also successfully infected, we have not found any specific symptoms known to or related to KSHV infection, such as skin lesions (Chang et al., 2009), B cell hyperplasia of humanized-BLT mice (Wang et al., 2014), and vascularized solid KS of nude mice (Mutlu et al., 2007). The possible explanation for this phenomenon is that immune-competent tree shrews were used in this study. However, clinical phenotypes often occurred in immune-deficient animals or coinfection with HIV patients. Another explanation is that it usually takes several years or decades for lethal disease symptoms to show after KSHV latency infection was established.

In this study, the KSHV infection was successfully established in TSKEC’s as an *in vitro* model and tree shrew animals as an *in vivo* model. Further, our results show that tree shrew is a more appropriate KSHV model than other animal models such as rodents. Thus, this small animal model is valuable for exploring the KSHV infection and may also help explain the cause of debilitating manifestations *in vivo* and a provide a valuable platform on which to characterize novel therapeutics against KSHV.

## Data Availability Statement

The original contributions generated for this study are included in the article/Supplementary Material, further inquiries can be directed to the corresponding authors.

## Ethics Statement

The animal study was reviewed and approved by Chinese Tree shrews (*Tupaia belangeri Chinensis*) (F1 generation) were obtained from the experimental animal center of the Kunming Institute of Zoology (KIZ), Chinese Academy of Sciences (CAS). All the animal-related experiments were strictly done in Animal Bio-safety level 2 laboratory according to the instruction of Chinese Regulations of Laboratory Animals (Ministry of Science and Technology of the People’s Republic of China) and Laboratory Animal—Requirements of Environment and Housing Facilities (GB 14925-2010, National Laboratory Animal Standardization Technical Committee). This study was reviewed and approved by the Experimental Animal Ethics Committee at Kunming University of Science and Technology.

## Author Contributions

All authors contributed to the concept of this study. XX, KL, and A-MZ designed the study. DL, ZB, YZ, LB, XW, and GW performed the study and acquired and analyzed data. XX, ZB, and DL wrote the manuscript. All authors reviewed and approved the final manuscript.

## Conflict of Interest

The authors declare that the research was conducted in the absence of any commercial or financial relationships that could be construed as a potential conflict of interest.

## Publisher’s Note

All claims expressed in this article are solely those of the authors and do not necessarily represent those of their affiliated organizations, or those of the publisher, the editors and the reviewers. Any product that may be evaluated in this article, or claim that may be made by its manufacturer, is not guaranteed or endorsed by the publisher.

## Supplementary Material

Supplementary Figure 1 Screening for rKSHV.219-susceptible infected primary cells from tree shrews. Primary cells were infected with rKSHV.219 (MOI = 10). Bright field, GFP and RFP fluorescence images were taken at 24 and 72 h after virus exposure. **(A)** HEK293T. **(B)** TSKEC. **(C)** TSH. **(D)** TSVEC. **(E)** TSPBMCs. **(F)** TSLEC. Original magnification, 10×.

Supplementary Figure 2 Bright field, GFP and RFP fluorescence images in the passaged TSKEC. Original magnification, 20×.

Supplementary Figure 3 Bright field and fluorescence microscopy images of rKSHV.219 in infected TSKEC and HEK293T. **(A)** TSKEC. **(B)** HEK293T. Original magnification, 20×.

Supplementary Figure 4 LANA and lytic protein ORF26 expressed in rKSHV.219-infected HEK293T.

Supplementary Figure 5 Immunofluorescence and differential interference contrast microscopy of rKSHV.219-infected PBMCs from TS1, TS5, and TS3 at 35, 49, and 119 dpi, respectively. It should be noted that those pictures showed an example of a rare positive field but not reflecting the overall incidence of GFP-positive cells. TS15 was the negative control (NC). Original magnification, 20×.

Supplementary Table 1 Primers for RT-PCR amplification.

Supplementary Table 2 GFP and RFP positive cells in rKSHV.219-inoculated cultures of TSKEC, TSH, TSVEC, TSPBMCs, TSLEC, and HEK293 at 48 h post-infection by flow cytometry.

Supplementary Table 3 GFP and RFP positive cells in rKSHV.219-inoculated TSKEC by flow cytometry.

Supplementary Table 4 GFP and RFP positive cells in rKSHV.219-inoculated HEK293T by flow cytometry.

Supplementary Table 5 Viral gene expression in PBMCs from rKSHV.219-infected tree shrews by RT-PCR analysis.

Supplementary Table 6 Tissue distribution of viral ORFK9 DNA in rKSHV.219-infected tree shrews.

Supplementary Table 7 Summary of rKSHV.219 infection in tree shrews.
