# Supplementary figures and images for: Establishment of Tree Shrew Animal Model for Kaposi’s Sarcoma-Associated Herpesvirus (HHV-8) Infection
Source: Front Microbiol. 2021 Sep 16;12:710067. doi: 10.3389/fmicb.2021.710067 (PMC8481836; doi:10.3389/fmicb.2021.710067)

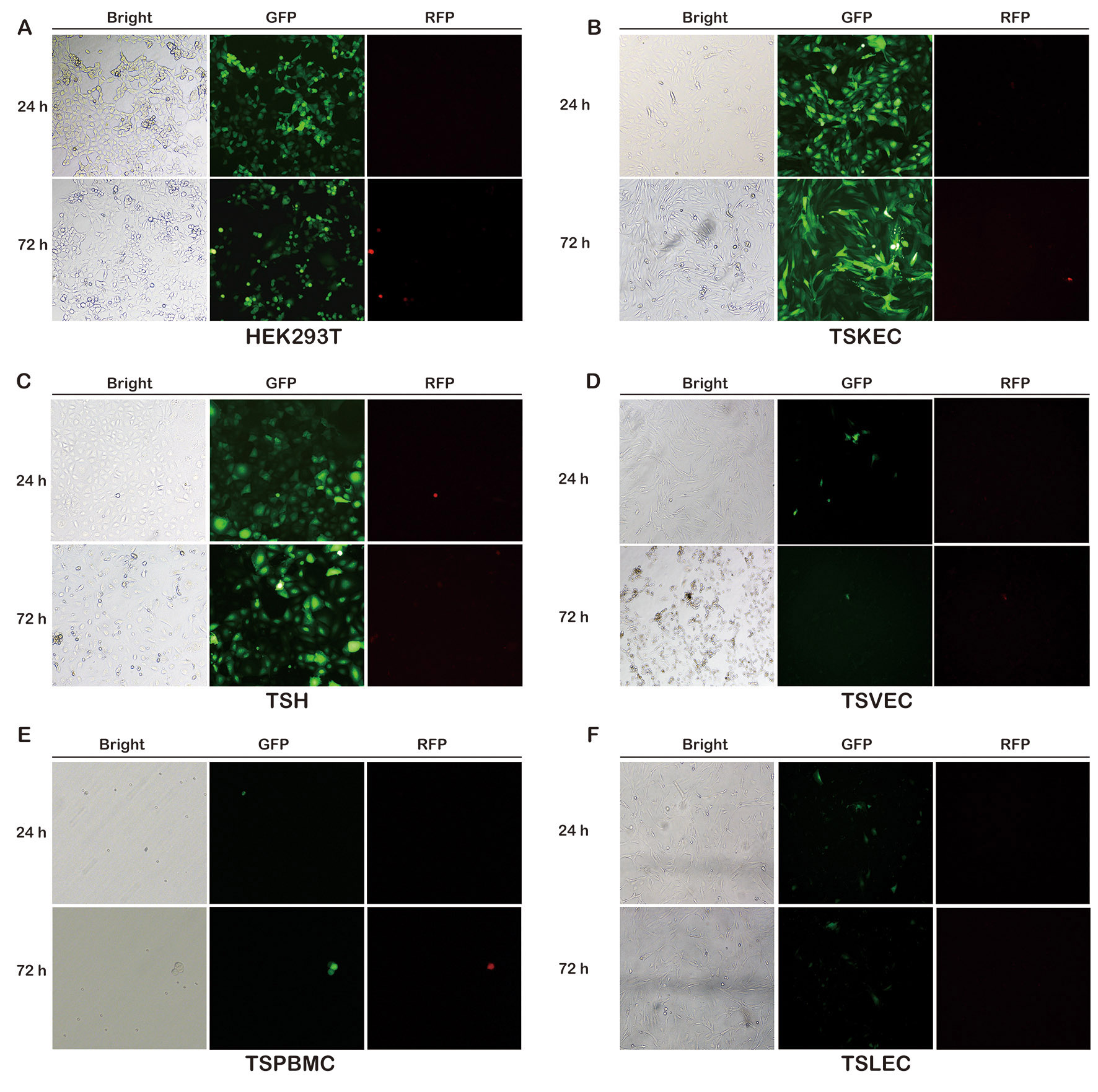

Supplement: Supplementary Figure 1 — Screening for rKSHV.219-susceptible infected primary cells from tree shrews. Primary cells were infected with rKSHV.219 (MOI = 10). Bright field, GFP and RFP fluorescence images were taken at 24 and 72 h after virus exposure. (A) HEK293T. (B) TSKEC. (C) TSH. (D) TSVEC. (E) TSPBMCs. (F) TSLEC. Original magnification, 10×. [file Image_1.JPEG]

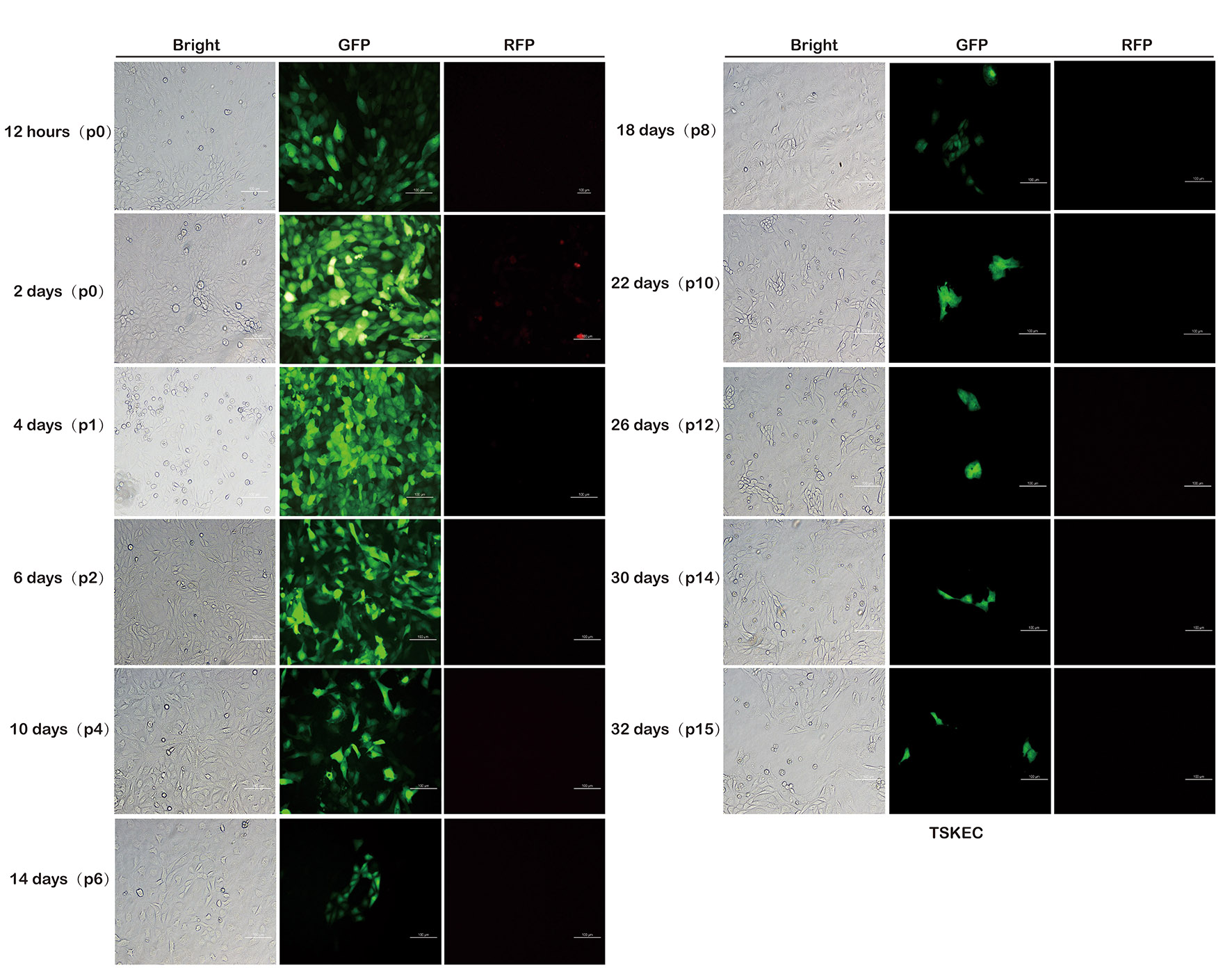

Supplement: Supplementary Figure 2 — Bright field, GFP and RFP fluorescence images in the passaged TSKEC. Original magnification, 20×. [file Image_2.JPEG]

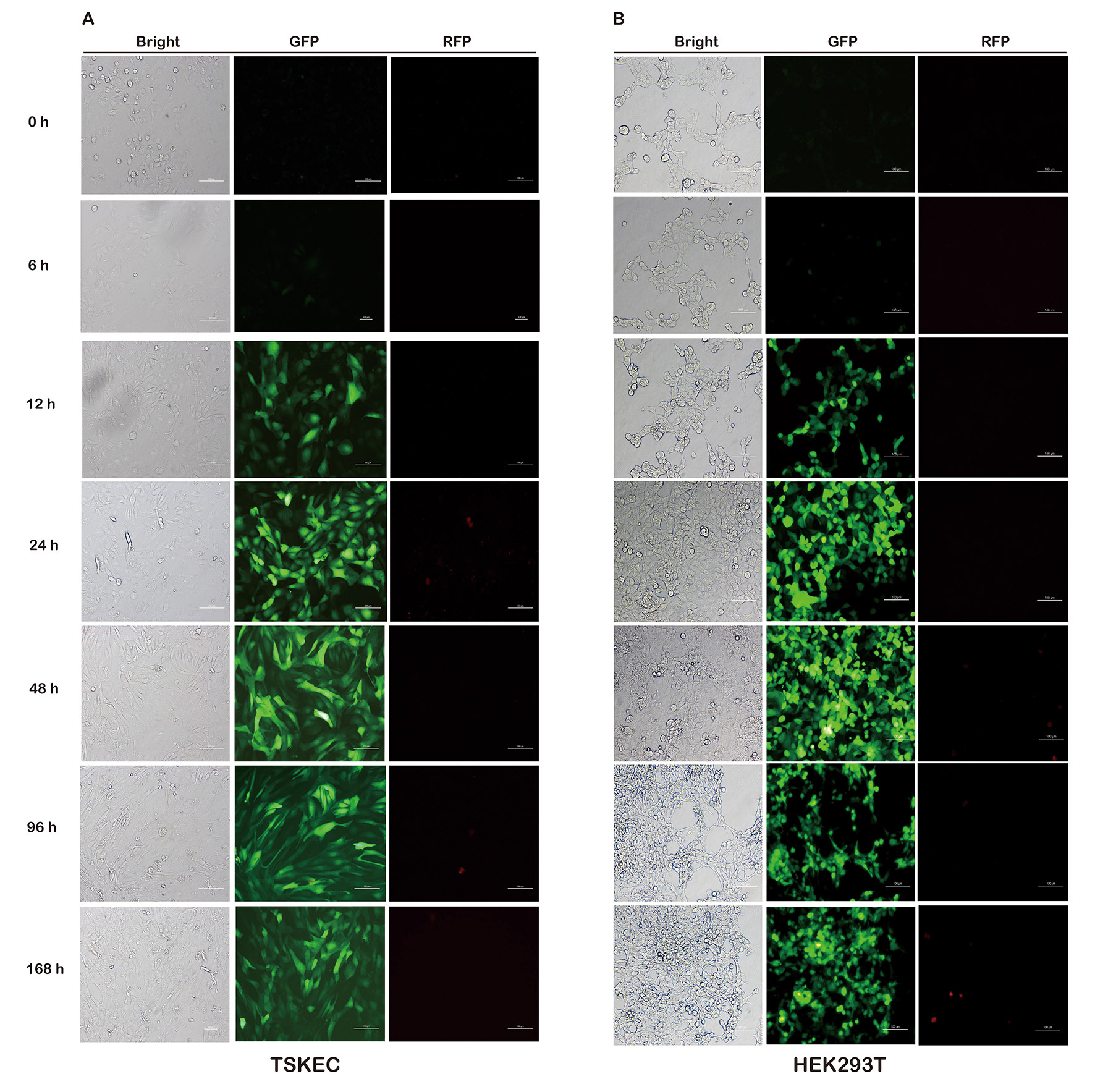

Supplement: Supplementary Figure 3 — Bright field and fluorescence microscopy images of rKSHV.219 in infected TSKEC and HEK293T. (A) TSKEC. (B) HEK293T. Original magnification, 20×. [file Image_3.JPEG]

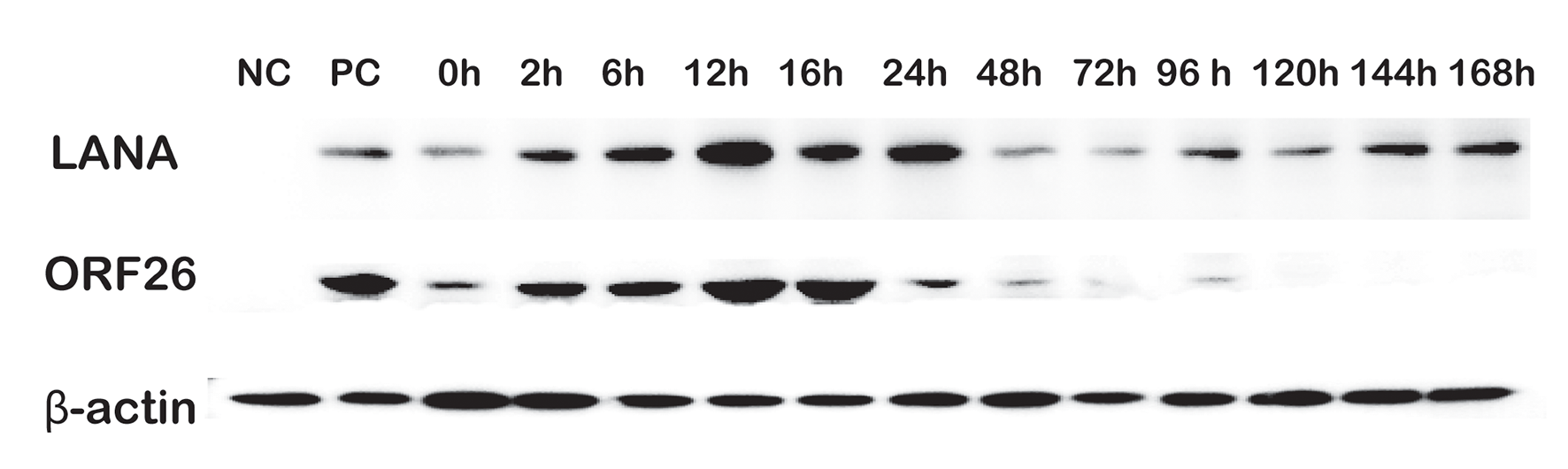

Supplement: Supplementary Figure 4 — LANA and lytic protein ORF26 expressed in rKSHV.219-infected HEK293T. [file Image_4.TIF]

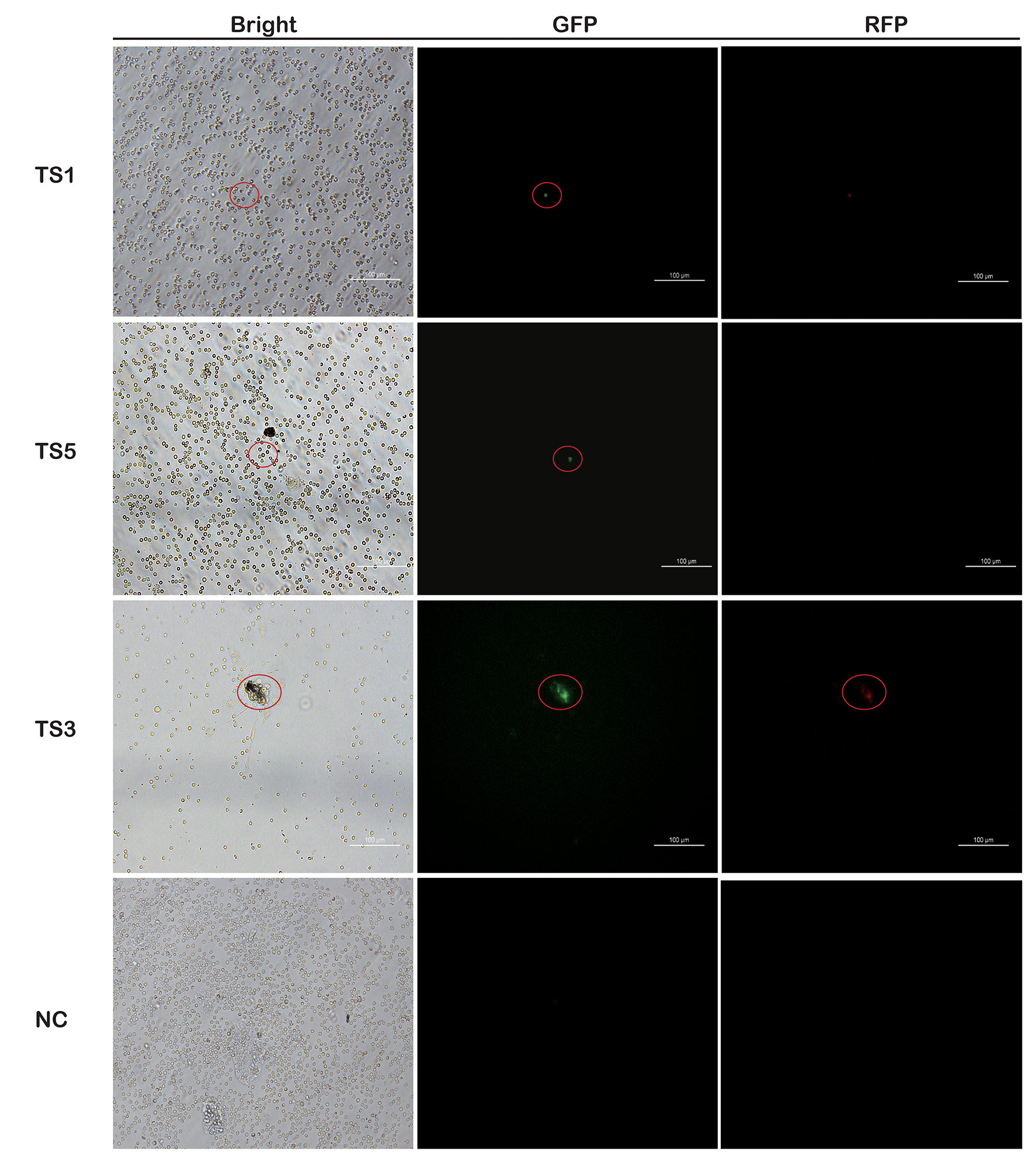

Supplement: Supplementary Figure 5 — Immunofluorescence and differential interference contrast microscopy of rKSHV.219-infected PBMCs from TS1, TS5, and TS3 at 35, 49, and 119 dpi, respectively. It should be noted that those pictures showed an example of a rare positive field but not reflecting the overall incidence of GFP-positive cells. TS15 was the negative control (NC). Original magnification, 20×. [file Image_5.TIF]
