# Supplementary material for: Establishment of Tree Shrew Animal Model for Kaposi’s Sarcoma-Associated Herpesvirus (HHV-8) Infection
Source: Front Microbiol. 2021 Sep 16;12:710067. doi: 10.3389/fmicb.2021.710067 (PMC8481836; doi:10.3389/fmicb.2021.710067)
Supplement: Supplementary Table 1 — Primers for RT-PCR amplification. [file Table_1.DOCX]

**Table S1.** Primers for RT-PCR amplification.

| Gene | Primer | Sequence (5'-3') | Annealing temperature (℃) |
| --- | --- | --- | --- |
| LANA | Forward | CCT CCA TCC CAT CCT GTG TC | 62 |
|  | Reverse | GGA CGC ATA GGT GTT GAA GAG |  |
| RTA | Forward | AGACCCGGCGTTTATTAGTACGT | 60 |
|  | Reverse | CAGTAATCACGGCCCCTTGA |  |
| ORF57 | Forward | TGGCGAGGTCAAGCTTAACTTC | 62 |
|  | Reverse | CCCCTGGCCTGTAGTATTCCA |  |
| ORF59 | Forward | TTG GCA CTC CAA CGA AAT ATT AGA A | 58 |
|  | Reverse | CGG GAA CCT TTT GCG AAG A |  |
| K8.1 | Forward | AAA GCG TCC AGG CCA CCA CAG A | 60 |
|  | Reverse | GGC AGA AAA TGG CAC ACG GTT AC |  |
| IL-1α | Forward | AGT TTC AGT CAG CCC ATC AC | 60 |
|  | Reverse | TTC ACA TTG CTT CGG GAG AG |  |
| IL-6 | Forward | CAG TCC AGT TGC CTT CTC | 60 |
|  | Reverse | GTC ACA TGC CTC TTG TTT C |  |
| IL-8 | Forward | CAC ATT CGA CGC CTT TTC ACC C | 60 |
|  | Reverse | TTT TCC TTG GGA TCC AGG CAG A |  |
| TNF-α | Forward | GCG TGC CAA CAC CCT CCT GA | 60 |
|  | Reverse | CAC AGG GGT GGA GGG GCA GC |  |
| TNF-β | Forward | CTT CGC GCT GAC CAA CAA | 60 |
|  | Reverse | GAG AAG ACC ACC TGC GAG TA |  |
| IFN-γ | Forward | AGT ATA CAA GTT ATA CAC TGG | 60 |
|  | Reverse | GTC ACT CTC CTC TGT CCA AT |  |
| β-actin (tree shrew) | Forward | GAGAGGGAAATCGTGCGTGAC | 58 |
|  | Reverse | CATCTGCTGGAAGGTGGACA |  |
