# Supplementary material for: Establishment of Tree Shrew Animal Model for Kaposi’s Sarcoma-Associated Herpesvirus (HHV-8) Infection
Source: Front Microbiol. 2021 Sep 16;12:710067. doi: 10.3389/fmicb.2021.710067 (PMC8481836; doi:10.3389/fmicb.2021.710067)
Supplement: Supplementary Table 2 — GFP and RFP positive cells in rKSHV.219-inoculated cultures of TSKEC, TSH, TSVEC, TSPBMCs, TSLEC, and HEK293 at 48 h post-infection by flow cytometry. [file Table_2.DOCX]

**Table S2.** GFP and RFP positive cells in rKSHV.219 inoculated cultures of TSKEC, TSH, TSVEC, TSPBMCs, TSLEC and HEK293 at 48 hours post-infection by flow cytometry.

| Cells  Fluorescence | TSKEC | TSH | TSVEC | TSPBMCs | TSLEC | HEK293T |
| --- | --- | --- | --- | --- | --- | --- |
| GFP (%) | 96.90±0.75 | 59.76±4.00 | 5.85±0.18 | (5.37±1.05)×10^-6^ | 2.05±0.31 | 62.77±4.18 |
| RFP (%) | 6.23±0.44 | 1.53±0.79 | 0.08±0.03 | (2.00±0.35)×10^-6^ | 0.03±0.02 | 4.22±0.12 |
