# Supplementary material for: Establishment of Tree Shrew Animal Model for Kaposi’s Sarcoma-Associated Herpesvirus (HHV-8) Infection
Source: Front Microbiol. 2021 Sep 16;12:710067. doi: 10.3389/fmicb.2021.710067 (PMC8481836; doi:10.3389/fmicb.2021.710067)
Supplement: Supplementary Table 3 — GFP and RFP positive cells in rKSHV.219-inoculated TSKEC by flow cytometry. [file Table_3.DOCX]

**Table S3.** GFP and RFP positive cells in rKSHV.219 inoculated TSKEC by flow cytometry.

| Time  Fluorescence | 12 h | 24 h | 48 h | 96 h | 168 h |
| --- | --- | --- | --- | --- | --- |
| GFP (%) | 93.27±0.40 | 97.1±0.26 | 96.9±0.75 | 96.53±0.84 | 69.71±0.93 |
| RFP (%) | 64.84±1.57 | 20.68±0.22 | 6.23±0.44 | 2.0±0.29 | 0.92±0.11 |
