# Supplementary material for: Establishment of Tree Shrew Animal Model for Kaposi’s Sarcoma-Associated Herpesvirus (HHV-8) Infection
Source: Front Microbiol. 2021 Sep 16;12:710067. doi: 10.3389/fmicb.2021.710067 (PMC8481836; doi:10.3389/fmicb.2021.710067)
Supplement: Supplementary Table 4 — GFP and RFP positive cells in rKSHV.219-inoculated HEK293T by flow cytometry. [file Table_4.DOCX]

**Table S4.** GFP and RFP positive cells in rKSHV.219 inoculated HEK293T by flow cytometry.

| Time  Fluorescence | 12 h | 24 h | 48 h | 96 h | 168 h |
| --- | --- | --- | --- | --- | --- |
| GFP (%) | 59.7±3.03 | 63.76±4.00 | 62.77±4.18 | 57.05±1.62 | 6.03±0.61 |
| RFP (%) | 14.26±6.00 | 7.59±1.29 | 4.22±0.12 | 3.88±0.45 | 0.05±0.02 |
