# Supplementary material for: Establishment of Tree Shrew Animal Model for Kaposi’s Sarcoma-Associated Herpesvirus (HHV-8) Infection
Source: Front Microbiol. 2021 Sep 16;12:710067. doi: 10.3389/fmicb.2021.710067 (PMC8481836; doi:10.3389/fmicb.2021.710067)
Supplement: Supplementary Table 5 — Viral gene expression in PBMCs from rKSHV.219-infected tree shrews by RT-PCR analysis. [file Table_5.DOCX]

| **Table S5.** Viral gene expression in PBMCs from rKSHV.219 infected tree shrews by RT-PCR analysis. | | | | | | | | | | | | | | | | | | |
| --- | --- | --- | --- | --- | --- | --- | --- | --- | --- | --- | --- | --- | --- | --- | --- | --- | --- | --- |
| Days  Gene | | 3 | 5 | 7 | 14 | 21 | | 28 | 35 | | 49 | 56 | | 63 | 77 | 91 | | 119 |
| TS1 | lana,  rta,orf57,  orf59,k8.1 | | lana,  orf57,orf59 | lana,  rta,orf57 | lana,  rta | | lana,  rta,orf57 | lana | | lana,  rta,orf59 | ND | | ND | ND | ND | | ND | ND |
| TS2 | lana,  rta,orf57,  orf59 | | lana,  rta,orf59,  k8.1 | lana | lana | | lana,  orf57,k8.1 | ND | | ND | ND | | ND | ND | ND | | ND | ND |
| TS3 | lana,  rta,orf57,  orf59,k8.1 | | lana,  rta,orf57,  orf59,k8.1 | - | - | | lana,  rta,k8.1 | - | | lana, | lana, | | lana, | lana, | lana,  rta, | | lana, | lana,  rta, |
| TS4 | lana,  rta, orf57,  orf59,k8.1 | | lana,  rta, orf57,  orf59 | - | - | | - | - | | lana, | - | | - | lana,  rta, | lana, | | lana,  rta,orf59 | ND |
| TS5 | lana,  rta, orf57,  orf59,k8.1 | | lana,  orf57,orf59,k8.1 | rta,  orf57 | - | | - | orf59 | | orf59 | lana,  orf59 | | ND | ND | ND | | ND | ND |
| TS6 | lana,  rta,orf57, orf59, k8.1 | | lana,  rta,orf57, orf59, | lana,  orf57,k8.1 | lana,  rta,orf57, orf59, | | ND | ND | | ND | ND | | ND | ND | ND | | ND | ND |
| TS7 | lana,  rta orf57,  orf59, | | lana,  orf57, | lana,  orf57, | orf59 | | - | orf59 | | orf59 | rta,  orf59 | | lana,  rta,orf57 | ND | ND | | ND | ND |
| TS8 | lana,  rta, orf57,  orf59,k8.1 | | lana,  rta, orf57,  orf59,k8.1 | orf57,  orf59 | ND | | ND | ND | | ND | ND | | ND | ND | ND | | ND | ND |
| TS9 | lana,  rta, orf57,  orf59,k8.1 | | lana,  rta, orf57,  orf59,k8.1 | orf57 | ND | | ND | ND | | ND | ND | | ND | ND | ND | | ND | ND |
| TS10 | lana,  rta,orf57,  k8.1 | | orf57 | lana,  orf57 | lana, | | lana, | - | | - | - | | lana, orf57,k8.1 | lana, orf57,k8.1 | ND | | ND | ND |
| TS11 | lana,  rta, orf57,  orf59,k8.1 | | lana,  rta, orf57,  orf59,k8.1 | lana,  rta | lana,  rta,orf57 | | ND | ND | | ND | ND | | ND | ND | ND | | ND | ND |
| TS12 | lana, rta,orf57, orf59,k8.1 | | lana, rta,orf57, orf59,k8.1 | lana, | lana, | | - | - | | lana, | - | | ND | ND | ND | | ND | ND |
| TS13 | lana, rta,orf57, orf59,k8.1 | | lana, rta,orf57, orf59,k8.1 | lana, | - | | - | ND | | ND | ND | | ND | ND | ND | | ND | ND |
| TS14 | - | | - | - | - | | - | ND | | ND | ND | | ND | ND | ND | | ND | ND |
| TS15 | - | | - | - | - | | - | - | | - | - | | - | - | ND | | ND | ND |
| TS16 | - | | - | - | - | | - | - | | - | - | | - | - | - | | - | - |

Note: −, negative; ND, no data.

The TS14, TS15 and TS16 tree shrews were as negative controls. They were killed on 21, 63 and 119 days, respectively.
