# Supplementary material for: Establishment of Tree Shrew Animal Model for Kaposi’s Sarcoma-Associated Herpesvirus (HHV-8) Infection
Source: Front Microbiol. 2021 Sep 16;12:710067. doi: 10.3389/fmicb.2021.710067 (PMC8481836; doi:10.3389/fmicb.2021.710067)
Supplement: Supplementary Table 6 — Tissue distribution of viral ORFK9 DNA in rKSHV.219-infected tree shrews. [file Table_6.DOCX]

**Table S6.** Tissue distribution of viral ORFK9 DNA in rKSHV.219-infected thirteen tree shrews.

| Tissues | TS1 | TS2 | TS3 | TS4 | TS5 | TS6 | TS7 | TS8 | TS9 | TS10 | TS11 | TS12 | TS13 | F* |
| --- | --- | --- | --- | --- | --- | --- | --- | --- | --- | --- | --- | --- | --- | --- |
| Spleen | **+** | **+** | **+** | **+** | **+** | **+** | **+** | **+** | **+** | **+** | **-** | **+** | **+** | 12 |
| Lung | **+** | **+** | **+** | **+** | **-** | **-** | **+** | **+** | **+** | **+** | **+** | **+** | **-** | 10 |
| Kidney | **-** | **-** | **+** | **+** | **-** | **-** | **+** | **+** | **-** | **-** | **-** | **-** | **+** | 5 |
| Liver | **+** | **-** | **+** | **-** | **-** | **+** | **+** | **-** | **-** | **-** | **-** | **+** | **-** | 5 |
| Thoracic aorta | **-** | **-** | **+** | **+** | **-** | **-** | **+** | **-** | **-** | **+** | **-** | **-** | **-** | 4 |

Note: F*, frequency. +, positive; −, negative.
