# Supplementary material for: Establishment of Tree Shrew Animal Model for Kaposi’s Sarcoma-Associated Herpesvirus (HHV-8) Infection
Source: Front Microbiol. 2021 Sep 16;12:710067. doi: 10.3389/fmicb.2021.710067 (PMC8481836; doi:10.3389/fmicb.2021.710067)
Supplement: Supplementary Table 7 — Summary of rKSHV.219 infection in tree shrews. [file Table_7.DOCX]

**Table S7.** Summary of rKSHV.219 infection in tree shrews.

| Number | KSHV copies | KSHV mRNA | HE | IHC | PBMCs | | Observation period | |
| --- | --- | --- | --- | --- | --- | --- | --- | --- |
| TS1 | + | + | - | + | + | 5 weeks (euthanasia) | |  |
| TS2 | + | + | - | - | - | 3 weeks (euthanasia) | |  |
| TS3 | + | + | + | + | + | 17 weeks (euthanasia) | |  |
| TS4 | + | + | + | + |  | 13 weeks (euthanasia) | |  |
| TS5 | + | + | + | + | + | 7 weeks (euthanasia) | |  |
| TS6 | + | + | - | - | - | 2 weeks (euthanasia) | |  |
| TS7 | + | + | + | + | - | 8 weeks (euthanasia) | |  |
| TS8 | + | + |  | + | - | 1 weeks (euthanasia) | |  |
| TS9 | + | + | + | + | - | 1 weeks (euthanasia) | |  |
| TS10 | + | + | + | + | - | 9 weeks (euthanasia) | |  |
| TS11 | + | + | + | + | - | 2 weeks (euthanasia) | |  |
| TS12 | + | + | - | - | - | 7 weeks (euthanasia) | |  |
| TS13 | + | + | + | - | - | 3 weeks (euthanasia) | |  |
| TS14 | - | - | - | - | - | 1 weeks (euthanasia) | |  |
| TS15 | - | - | - | - | - | 7 weeks (euthanasia) | |  |
| TS16 | - | - | - | - | - | 17 weeks (euthanasia) | |  |

Note: +, positive. −, negative. PBMCs, Green fluorescent cells of KSHV infection in peripheral blood lymphocytes of tree shrews.

The TS14, TS15 and TS16 tree shrews were as negative controls.
